# Lytic enzyme production optimization using low-cost substrates and its application in the clarification of xanthan gum culture broth

**DOI:** 10.1002/fsn3.87

**Published:** 2014-04-25

**Authors:** Cíntia Reis da Silva, Marilia Lordelo Cardoso Silva, Helio Mitoshi Kamida, Aristoteles Goes-Neto, Maria Gabriela Bello Koblitz

**Affiliations:** 1 State University of Feira de SantanaAv. Transnordestina, Novo Horizonte, Feira de Santana, 44036-900, Bahia, Brazil; 2 Federal University of the Rio de Janeiro StateAv. Pasteur, 296 - Bloco II, Urca, Rio de Janeiro, 22290-240, Rio de Janeiro, Brazil

**Keywords:** *Aspergillus tamarii*, gram-negative bacteria, protease, response surface methodology

## Abstract

Lytic enzymes are widely used in industrial biotechnology as they are able to hydrolyze the bacterial cell wall. One application of these enzymes is the clarification of the culture broth for the production of xanthan gum, because of its viability in viscous media and high specificity. The screening process for filamentous fungi producing lytic enzymes, the optimization of production of these enzymes by the selected microorganism, and the optimization of the application of the enzymes produced in the clarification of culture broth are presented in this article. Eleven fungal isolates were tested for their ability to produce enzymes able to increase the transmittance of the culture broth containing cells of *Xanthomonas campestris*. To optimize the secretion of lytic enzymes by the selected microorganism the following variables were tested: solid substrate, initial pH, incubation temperature, and addition of inducer (gelatin). Thereafter, secretion of the enzymes over time of incubation was assessed. To optimize the clarification process a central composite rotational design was applied in which the pH of the reaction medium, the dilution of the broth, and the reaction temperature were evaluated. The isolate identified as *Aspergillus tamarii* was selected for increasing the transmittance of the broth from 2.1% to 54.8%. The best conditions for cultivation of this microorganism were: use of coconut husk as solid substrate, with 90% moisture, at 30°C for 20 days. The lytic enzymes produced thereby were able to increase the transmittance of the culture broth from 2.1% to 70.6% at 65°C, without dilution and without pH adjustment.

## Introduction

Lytic enzymes present a range of applications in industrial biotechnology: in controlling the development of food pathogens (Oliveira et al. 2012), in the release of enzymes from lactic acid bacteria to cheese ripening, and in the release of other intracellular products such as polyhydroxybutyrate produced by *Bacillus* sp. (Loessner 2005). The lytic enzymes, also called lysozymes, lysines or murein hydrolases, are able to hydrolyze the peptidoglycan responsible for the rigidity of the bacterial cell wall (Fischetti 2010).

Lytic enzymes are produced by a variety of microorganisms, not only viruses (bacteriophages; Loessner 2005; Salazar and Asenjo 2007; Fischetti 2010; Oliveira et al. 2012) but also bacteria (especially lactic acid bacteria; Ohbuchi et al. 2001; Lortal and Chapot-Chartier 2005). Although lytic enzymes of bacterial and viral origin are well described in the literature (Ohbuchi et al. 2001; Loessner 2005; Lortal and Chapot-Chartier 2005; Salazar and Asenjo 2007; Fischetti 2010; Oliveira et al. 2012), little attention has been given to the ability of filamentous fungi to secrete enzymes capable of lysing the cell wall of gram-negative bacteria.

The bacteria can be divided into two groups, gram-positive and gram-negative bacteria, based on the characteristics of their cell wall. While gram-positive bacteria exhibit cell wall composed of multiple layers of peptidoglycan associated with amino acids and their derivatives, on gram-negative bacteria the peptidoglycan layer is much thinner and covered by an outer membrane composed of proteins, phospholipids, lipoproteins, and lipopolysaccharides. The presence of this membrane increases the resistance of gram-negative bacteria to lysis by murein hydrolases and to attack by bacteriocins. The lysis of their cell walls depends on the application of auxiliary processes for removing the outer membrane, such as the use of detergents or combination with amidases and proteases (Salazar and Asenjo 2007).

Xanthan gum, an extracellular polysaccharide secreted by *Xanthomonas campestris*, a gram-negative bacterium, is a widely used gelling agent, viscosity controller, and lubricant (Psomas et al. 2007; Silva et al. 2009). For any of these applications, bacterial cells must be removed from the fermented broth. The nature of the product causes the culture broth to present a very high viscosity, which precludes separation of the cell mass by conventional methods like filtration or centrifugation. Alternatives, such as use of extreme pH values (acid or alkaline), are not only effective in the destruction of the cells but can also damage the polysaccharide causing decrease in its viscosity. An alternative methodology, considered highly promising, is the use of lytic enzymes for removal of the cell wall of bacteria. Enzyme activity has the advantage of being applicable in viscous media, show high specificity (causing little or no damage to the gum present) and cause cell lysis, preventing the survival of the bacterium in question (Triveni and Shamala 1999; Shastry and Prasad 2002).

This study aimed to select filamentous fungi producing lytic enzymes, optimize the production of these enzymes in low-cost substrate, and to optimize the application of enzymes produced in the clarification of the culture broth of *X. campestris* pv. *campestris* applied for the biotechnological production of xanthan gum. As a result, it is expected to provide a low-cost alternative for clarification of xanthan gum that can be produced in the same industrial plant and renders the final product price competitive.

## Material and Methods

### Microorganisms

An isolate of *X. campestris* pv. *campestris* 247, belonging to the Section of Phytopathological Bacteriology of the Culture Collection of the Biological Institute of Campinas/SP, Brazil, was used for the production of the broth to be clarified. For the production of lytic enzymes, filamentous fungi belonging to the Culture Collection of Microorganisms from Bahia (CCMB) and the UEFS Herbarium (HUEFS) were tested.

The strain of *X. campestris* was maintained in yeast–malt (YM) standard medium consisting of (% w/v) 0.3% yeast extract, 0.3% malt extract, 0.5% peptone, 1.0% glucose, and 2.5% agar (for solid medium; Haynes et al. 1995). The inoculated strains were maintained in an incubator at 28 ± 2°C for 24 h and, after this period, stored at 4 ± 1°C. Subcultures were made every 30 days. All procedures were performed aseptically.

Eleven isolates of filamentous fungi were transferred to Petri dishes containing potato dextrose agar standard medium, were maintained in an incubator for 8 days at 28 ± 2°C, and after this period, stored at 4 ± 1°C. Subcultures were performed every 90 days. All procedures were performed aseptically.

### Production of the culture broth containing cells of X. campestris (Rottava et al. 2009)

The production of the broth was performed in three steps. (1) First a pre-inoculum was prepared: three loops full of cell mass grown on YM agar were inoculated into 250 mL Erlenmeyer flasks containing 50 mL of liquid YM medium. This medium was incubated in an orbital shaker at 120 rpm, 28 ± 2°C, until reaching an optical density at 560 nm (OD_560_) of about 2.0, which occurred after 22–26 h of incubation. (2) After this period, the inoculum was prepared by aseptically transferring 1 mL of the pre-inoculum to 250 mL Erlenmeyer flasks containing 50 mL of liquid YM medium. This medium was incubated under the same conditions described above until OD_560_ ranging from 2.5 to 5.5 was achieved, which occurred after 40 h of incubation, and corresponded to a cell concentration of 10^11^ CFU (colony-forming units)/mL. (3) Finally, 14 mL of this inoculum was aseptically transferred to 86 mL fermentation medium consisting of (% w/v): 0.02% MgSO_4_·7H_2_O, 0.5% KH_2_PO_4_, 0.0006% H_3_BO_3_, 0.2% (NH_4_)_2_SO_4_, 0.00024% FeCl_3_, 0.0002% CaCl_2_·2H_2_O, 0.0002% ZnSO_4_, 0.20% citric acid, 5% sucrose, and pH = 7.0. The medium was incubated in an orbital shaker at 120 rpm, 28 ± 2°C for 72 h. The bacterial viscous broth obtained was pasteurized at 70°C for 30 min and kept at −18 ± 2°C until use. This broth showed transmittance (%) of 2.1 ± 1.0 at 540 nm.

### Selection of strains according to their lytic activity

#### Production of lytic enzymes

For the production of lytic enzymes from filamentous fungi, five plugs (6 mm diameter) of solid medium containing mycelia (with or without spores) after 8 days of growth were applied. This inoculum was aseptically transferred to 500 mL Erlenmeyer flasks containing 50 mL of medium consisting of (% w/v) 0.7% (NH_4_)_3_PO_4_, 0.15% K_2_HPO_4_, 0.05% MgSO_4_·7H_2_O, 0.03% CaCl_2_, 0.25% trace salt solution (0.1% FeSO_4_, 0.1% MnCl_2_·4H_2_O, 0.1% ZnSO_4_·7H_2_O in 100 mL of distilled water), 0.5% gelatin, and pH = 7.0. The fungi were maintained, without stirring, in an incubator at 28 ± 2°C for 8 days. After the incubation period, the culture medium was filtered into previously sterilized gauze. The filtrate, called crude enzyme extract, was aliquoted and stored at −18 ± 2°C until use.

#### Evaluation of lytic enzymes in the clarification

For evaluation of lytic enzymes in the clarification of broth culture containing gram-negative bacteria, a factorial experimental design (2^2^) was applied, testing two independent variables at three levels: pH (4.0, 7.0, and 10.0) and temperature (20, 40, and 60°C). The assays were performed according to the spreadsheet generated by the software Statistica (7.0) as shown in Table 1. As dependent variable (response), the clarification efficiency of the enzymes on culture broth was measured according to the lytic activity described as follows.

**Table 1:** Lytic capacity of the crude enzymatic extract produced by different fungal isolates on culture broth of *Xanthomonas campestris*

			%T (540 nm)										
			
Assay	Temperature (°C)	pH	Asp32	Asp39	Asp	F	G	I	FX 127	N	O	P	T
1	20	4.0	4.2	2.1	9.1	8.2	12.7	8.3	11.5	9.5	7.3	4.4	5.5
2	60	4.0	0	3.6	54.8	15.8	7.0	0	8.5	5.1	2.1	4.8	4.5
3	20	10.0	18	26.2	29.6	13.2	29.3	51.7	22.2	33.7	16.0	49.3	47.6
4	60	10.0	51.7	49.4	50.1	44.4	18.9	14.1	13.7	8.2	8.5	11.5	0.3
5	40	7.0	0	0.1	30.7	10.1	38.2	2.1	50.0	47.3	57.0	0	0
6	40	7.0	3.1	0	36.7	2.4	40.5	1.0	49.2	49.6	48.8	0	0.4
7	40	7.0	4.9	0	36.6	3.2	43.1	1.5	42.0	46.4	49.8	0	0.2
*8*	40	7.0	3.1	0	32.5	3.2	41.6	1.1	42.4	51.1	46.3	0	0

Initial %T of the broth = 2.1 ± 1.0.

The lytic activity was tested according to the modified methodology of Shastry and Prasad (2002). In assay tubes, the bacterial broth culture (*X. campestris*) was diluted (1:1) with buffer at the pH value indicated in Table 1 and 2 mL of crude enzyme extract was added. The mixture was kept at the temperature indicated in Table 1, in a static bath for a period of 24 h. The transmittance was evaluated at 540 nm against a blank, where water was added in place of enzyme extract. The lytic activity was expressed as transmittance (%) at 540 nm of the treated mixture. The closer to 100% this value, the greater the activity of the lytic enzyme crude extract tested.

#### Evaluation of the best conditions for the production of lytic enzymes

To determine the best growing conditions that favored the secretion of lytic enzymes and to assess the effects of independent variables on the secretion of protease activity by the selected microorganism, a strategy of sequential statistical designs was used in this work, according to the spreadsheets generated by the software Statistica (7.0). To evaluate the influence of the four variables under study: incubation temperature (20–40°C), initial pH value (4.0–11.0), solid substrate (coconut fiber and sugarcane bagasse), and presence of inducer (gelatin 0–2%) in the response of enzyme activity (U/mL), an exploratory experiment was conducted applying a fractional factorial experimental design (2^4−1^), according to the assay spreadsheet presented in Table 2. After analyzing the effects of four independent variables, a central composite rotational design (DCCR) was conducted with the aim of optimizing the variables temperature (10.0–50.0°C), initial pH value (3.0–11.0), and the addition of inductor (0–5% gelatin) for maximum secretion of proteases, including six axial points and four center point replicates, totaling 18 assays.

**Table 2:** Matrix of the experimental design (2^4−1^) and response in enzymatic activity (U/mL)

Assay	pH	Temperature (°C)	Substrate (1 + 2)	Gelatin (%)	Activity (U/mL)
1	3.0	20	0 + 100	0	270.00
2	11.0	20	0 + 100	2	2,876.67
3	3.0	40	0 + 100	2	2,123.33
4	11.0	40	0 + 100	0	470.00
5	3.0	20	100 + 0	2	243.33
6	11.0	20	100 + 0	0	63.33
7	3.0	40	100 + 0	0	83.33
8	11.0	40	100 + 0	2	2,646.67
9(C)	7.0	30	50 + 50	1	2,443.33
10(C)	7.0	30	50 + 50	1	2,643.33
11(C)	7.0	30	50 + 50	1	2,403.33
12(C)	7.0	30	50 + 50	1	2,116.67

1, % sugarcane bagasse; 2, % coconut fiber.

The culture medium, composed of 2 g of dry solid substrate moistened with 18 g buffer at the indicated pH value (90% moisture) in 500 mL Erlenmeyer flasks, was added with the inoculum (five plugs of solid medium containing mycelium and spores) and kept in an incubator at the indicated temperature for 8 days. After the incubation period, the medium was added with 50 mL of distilled water at 4°C and kept on ice for 1 h with occasional shaking. This mixture was then filtered into previously sterilized gauze. The filtrate, called crude enzyme extract, was aliquoted and stored at −18 ± 2°C until use.

### Determination of proteolytic activity

The proteolytic activity was evaluated according to the method of Kunitz (1974), modified by Walter (1984) and Dienes et al. (2007), using a solution of casein (2.0%, pH = 6.0) as substrate. The reaction medium consisted of 1.5 mL of substrate solution and 1.0 mL of phosphate buffer (0.1 mol/L, pH 7.0). The medium remained in the bath at 40°C for 5 min and then was added with an aliquot of 0.5 mL of the crude enzyme extract initiating the reaction. The reaction medium was maintained at 40°C for 30 min and the reaction was stopped by adding 3.0 mL of trichloroacetic acid (TCA 0.4 mol/L). An aliquot of 1.5 mL of this medium was transferred to a microcentrifuge tube and centrifuged at 13,600*g* for 15 min. The supernatant was measured in a spectrophotometer at 280 nm against a blank in which the enzyme extract was added just after addition of TCA. One unit of activity was defined as the amount of enzyme required to raise absorbance in 0.001.

### Time course for enzymatic secretion

To evaluate the effect of incubation time in the secretion of the enzymes, the five plugs (6 mm diameter) of solid medium containing mycelium with spores were transferred into 500 mL Erlenmeyer flasks containing 20 g of coconut fiber (pH 7.0, 2.5% gelatin, and 90% humidity) and incubated at 30°C. Samples in triplicate were analyzed for the production of lytic enzymes every 5 days in a maximum time of 30 days. Data were subjected to analysis of variance followed by the Scott-Knott test, at 5% probability. Analyses were performed by free software SISVAR (5.0).

### Application of enzymes produced in the clarification of the culture broth of *X. campestris*

To determine the best process conditions for clarification of xanthan gum by the crude enzyme, a proteolytic activity of 3200 U was fixed. A DCCR was conducted according to the assay spreadsheet presented in Table 3. The variables evaluated were: concentration of the culture broth (10–90%), temperature (20–80°C), and pH (3.0–11.0). As dependent variable (response), the transmittance (%) of the clarified broth was measured.

**Table 3:** Optimization of the clarification process of *Xanthomonas campestris* culture broth by lytic enzymes from *Aspergillus tamarii*

Assay	pH	Temperature (°C)	Culture broth	Transmittance (%)
1	4.6	32	26	35.72
2	9.4	32	26	28.79
3	4.6	68	26	52.41
4	9.4	68	26	47.32
5	4.6	32	74	38.47
6	9.4	32	74	31.43
7	4.6	68	74	68.71
8	9.4	68	74	57.32
9	3.0	50	50	54.37
10	11.0	50	50	48.74
11	7.0	20	50	15.72
12	7.0	80	50	21.85
13	7.0	50	10	43.21
14	7.0	50	90	64.87
15(C)	7.0	50	50	52.34
16(C)	7.0	50	50	51.71
17(C)	7.0	50	50	53.40
18(C)	7.0	50	50	53.81

Initial %T of the broth = 2.1 ± 1.0.

## Results and Discussion

### Screening of strains according to their lytic activity

**Figure 1:**
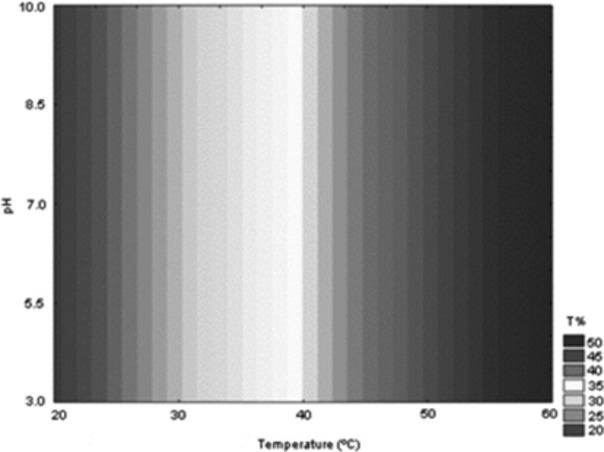
Fitted response profile (*r*² = 0.9290) for the lytic activity of *Aspergillus tamarii* as a function of temperature and reaction medium pH. 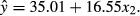

Most studies involving screening for enzymatic activity evaluate this activity in fixed conditions of temperature and pH (Rojas et al. 2009; Cruz Ramírez et al. 2012), since testing different combinations may lead to the need for a very large number of tests. However, tests on fixed conditions may disregard the enzymatic activity manifested in conditions different from those tested. In this study, we used a factorial statistical design (2^2^), which allowed the evaluation of lytic activity in different combinations, involving three levels of temperature and three pH values, enabling check the lytic ability of isolates in different reaction conditions, totaling eight tests for each isolate (Table 1). The statistical design also allowed the generation of a response surface capable of showing, among the conditions tested, those which presented more satisfactory results (Fig. 1).

The evaluation of the results regarding the increase in transmittance after the lytic activity on the culture broth containing cells of *X. campestris* (Table 1) showed that all isolates were able to, in some of the conditions tested, causing an increase in transmittance of the broth, although isolated Asp, Asp 32, and I have showed better results. Of these three isolates, Asp presented the best results of the final transmittance (54.8%). This isolate was then selected for further testing and was identified as *Aspergillus tamarii*. Hieda et al. (1991), in a paper about screening for fungi able to secrete lytic enzymes on gram-positive and gram-negative bacteria, also selected two species of *Aspergillus* as best secretors of the enzymes of interest.

According to Triveni and Shamala (1999) and Shastry and Prasad (2002), the main lytic activity responsible for the clarification of the culture broth containing cells of *X. campestris*, a gram-negative bacterium, is the proteolytic activity. As a consequence, to improve the lytic activity of the crude enzyme extract of *A. tamarii*, this study sought to optimize the growth conditions of the fungus to ensure the greatest possible secretion of proteases. According to Rao et al. (1998), a single species of fungus can produce different isoforms of proteases (alkaline, neutral, and acid). *Aspergillus tamarii* proved capable of producing enzymes active in a broad pH range, which makes it a potentially promising microorganism for the production of proteolytic enzymes in the lysis of gram-negative bacteria, in different reaction conditions. The analysis of the response surface generated (Fig. 1) showed that transmittance values above 40% could be achieved with treatments in any pH value, provided that the reaction temperature was above 45°C; however, for superior results (above 50% transmittance), treatments should be applied at temperatures above 60°C. High optimum temperature of activity is a quite interesting characteristic, since, after the fermentation of the medium by *Xanthomonas*, the broth must be subjected to heat treatment in order to: inactivate the microorganism which is pathogenic; to increase the removal of xanthan gum from the cells, inactivate enzymes, and to improve the rheological properties of the gum (García-Ochoa et al. 2000). The use of lytic enzymes that show optimum activity at high temperatures can allow its addition during thermal processing, reducing the total duration of the process.

### Evaluation of the best conditions for the production of lytic enzymes

#### Exploratory experiment

For enzyme production by filamentous fungi, the solid substrate fermentation system is very interesting, since it is a simple procedure, inexpensive, that enables easy recovery of extracellular enzymes secreted, besides facilitating development of these microorganisms by promoting the colonization of the medium surface, ensuring the desired oxygenation, without stirring, that could lead to disruption of hyphae (Vishwanatha et al. 2010). According to Hajji et al. (2008), the secretion of proteases by microorganisms is directly influenced by the composition of the culture medium and by physical factors such as incubation temperature. The medium composition is of particular importance for enzymes for industrial use since it represents 30–40% of the total production cost. Cost reduction can be achieved by optimization of enzyme secretion and the use of low-cost components, such as agro-industrial wastes. To warrant an efficient optimization, several studies have been applying multifactorial designs of experiments, based on the assessment of response surface, which has already proved successful in various areas of biotechnology (Thanapimmetha et al. 2012).

**Figure 2:**
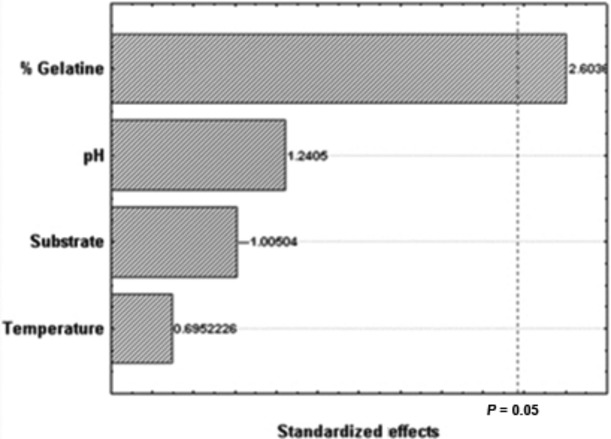
Pareto chart of effects (95% significance) for the exploratory experiment on the evaluation of the best conditions for the production of lytic enzymes by *Aspergillus tamarii*.

In this study, we tested two different low-cost substrates, abundant in northeastern Brazil (sugarcane bagasse and coconut fiber). Initially, an exploratory experiment was conducted in order to verify that all factors tested had real influence on the secretion of proteases by *A. tamarii* and to evaluate the orders of magnitude of the parameters under evaluation. The results of this experiment are shown in Table 2 and Figure 2.

According to the results, the variable incubation temperature was not significant (Fig. 2) to increase the proteolytic activity secreted by *A. tamarii*. However, it has been hypothesized that the temperature range tested in this case was very narrow (20–40°C) and that temperature would possibly show significance if assessed in a wider range. Thus, this variable was kept in the following experiment, and the range between 10 and 50°C was tested.

The variable initial pH of the reaction medium was also not significant (Fig. 2). This may be a consequence of the fact that a wide pH range is able to provide the ideal conditions for the secretion of proteases (Fig. 1). Indeed, there was a clear indication that the best results could be obtained at pH values between 7.0 and 11.0, similar to that found by Anandan et al. (2007), when working with the same species, and also by Hajji et al. (2008), when working with other species of the same genus (*A. clavatus*). However, since the pH of the culture medium can affect various enzymatic processes such as protein synthesis and transport across membranes (Anandan et al. 2007) and that the initial pH of the medium was considered as a critical variable by Vishwanatha et al. (2010) working with *Aspergillus oryzae*, and by Ravikumar et al. (2012) working with *Pleurotus sajor-caju*, it was considered wise to keep this variable in the experiment that followed. The variable solid substrate was not considered significant (Fig. 2), in disagreement with the results obtained by Vishwanatha et al. (2010) and by Ravikumar et al. (2012). However, in these two cases, the solid substrate selected was the one providing the highest protein content to the reaction medium, less important characteristic in the present experiment, which used supplementation of the medium with gelatin for induction of secretion of proteases, which was considered the variable of greater influence. According to Geisseler and Horwath (2008), the presence of proteins in the culture medium can induce the secretion of proteases in various microorganisms. Furthermore, the increase in enzyme activity was influenced by the concentration of protein added. In this work, the tested protein was gelatin, which is a solubilizable protein of relatively low cost. Therefore, for subsequent testing, green coconut fiber was selected as substrate, being a product with less variety of applications than the sugarcane bagasse and that poses disposal issues in many tropical coastal regions. The maximum concentration of gelatin tested was increased to 5% in an attempt to verify the effect of excess gelatin in the secretion of proteases by the strain of *A. tamarii* under study.

### Optimization

Table 4 shows that the regression was significant (*P* < 0.10) and that the calculated *F* value was higher than the tabulated *F* value in 1% level of significance. The value of *r*² indicates that a percentage of 60.44% of the variation is explained by the model, and that this model is appropriate to assess the influence of the significant variables on the production of the enzyme under study.

**Table 4:** Variance analysis for the secretion of protease by *Aspergillus tamarii*

Variation source	SS	df	MS	*F*	Significance
Regression	4295689.37	3	1431896	7.130156	*P* < 0.10
Residue	2811516.33	14	200822.6		
Lack of fit	2811283	11			
Pure error	233	3			
Total	7107206	17			

*r*² = 0.6044; *F*_3,14,0,01_ = 5.56. SS, square sum; df, degrees of freedom; MS, mean square.

**Figure 3:**
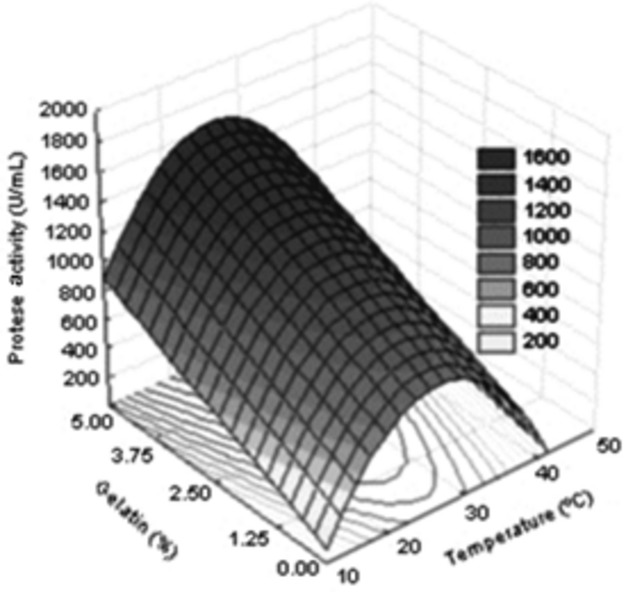
Fitted response surface (*r*² = 0.6044) for protease activity secreted by *Aspergillus tamarii*: temperature (*X*_2_) versus percentage of gelatin (*X*_3_). 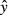 = 1197.197-288.420 x_2_-420.218 x_2_^2^ + 234.551 x_3_.

Figure 3 illustrates the influence of temperature and addition of gelatin in the secretion of proteases by *A. tamarii*, using coconut fiber as the substrate. The variable temperature showed significant quadratic effect in this experiment (*P* < 0.01), confirming the previous hypothesis that this influence was not detected by the exploratory experiment because of the narrow range of values tested. The temperature optimum ranged between 20 and 35°C, in agreement with the results obtained by Anandan et al. (2007), Hajji et al. (2008), Vishwanatha et al. (2010), and Thanapimmetha et al. (2012), confirming that the secretion of proteases by various species of *Aspergillus*, including *A. tamarii*, is higher at a temperature of 30°C and decreases at higher and lower temperature values. According to Anandan et al. (2007), the incubation temperature has greater influence on processes of solid-state fermentation than on submerged fermentation. This effect is related not only to the regulation of enzyme synthesis but also the secretion of enzymes by the permeabilization of the cell wall. According to Hajji et al. (2008), most filamentous fungi present optimum temperature for enzyme secretion between 28 and 30°C.

The variable concentration of gelatin showed a significant linear effect (*P* < 0.10), but no significant quadratic effect, indicating that within the tested range, the higher the supplementation of gelatin in the medium, the greater must be the secretion of proteases by the fungus, in agreement with the work of Geisseler and Horwath (2008). However, the increase in activity achieved by increasing the gelatin concentration was not enough to justify the increased cost of supplementation because when the concentration of gelatin was doubled from 2.5% to 5.0%, the increase in the protease activity was only 8.55%, from 1512.67 to 1632.00 U/mL.

In accordance with the results, the optimized conditions for the synthesis of proteolytic enzymes by the selected strain of *A. tamarii* were: coconut fiber substrate, 90% moisture, pH 7.0, added with 2.5% gelatin at 30°C.

### Time course for enzymatic secretion

**Figure 4:**
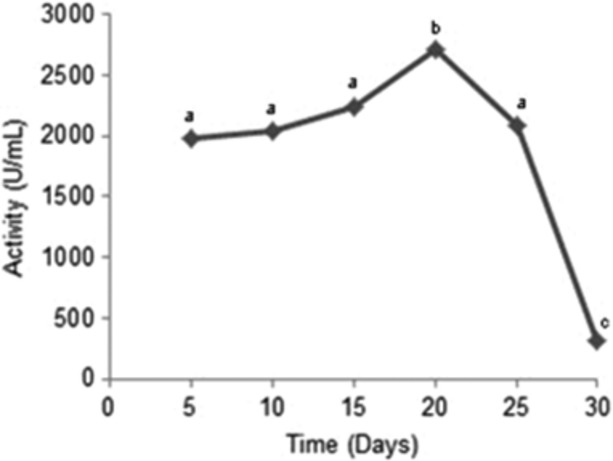
Secretion of proteolytic enzymes by *Aspergillus tamarii* as a function of incubation time.

As can be observed in Figure 4, the maximal secretion of proteolytic enzymes (2720.00 U/mL, 68,000 U/g) occurred after a 480 h (20 days) incubation period. This time was significantly longer than that required for maximal secretion of protease in other experiments: Thanapimmetha et al. (2012) 96 h, 3094 U/g; Vishwanatha et al. (2010) 96 h, 1600 U/g and Hajji et al. (2008) 72 h, 788 U/mL. However, compared to these same studies, the activity secreted by *A tamarii* this work was also significantly higher. Moreover, although maximal secretion has been reached after a long incubation period, significant amounts of activity were produced even after 120 h (1980 U/mL, 49,500 U/g).

### Application of enzymes produced in the clarification of the culture broth of *X. campestris*

**Figure 5:**
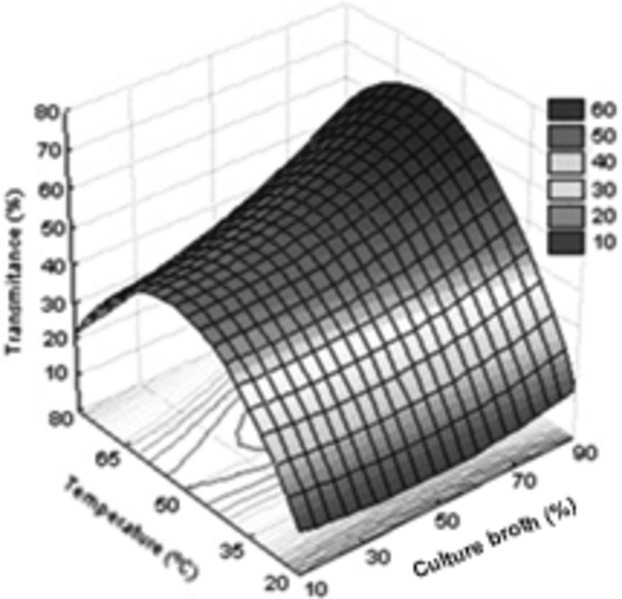
Fitted response surface (*r*² = 0.8258) for the clarification of the culture broth containing lytic enzymes of *Aspergillus tamarii*: reaction temperature (*X*_2_) versus concentration of culture broth (*X*_3_). 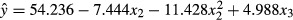.

From the variance analysis shown in Table 5, the model describing the clarification of the culture broth as a function of its dilution and the temperature of reaction was considered adequate. The percentage of variance explained is 82.58% and the value of calculated *F* is significant (*P* < 0.001), satisfying the conditions for the construction of the response surface shown in Figure 5.

**Table 5:** Variance analysis for the clarification of the broth of *Xanthomonas campestris* by lytic enzymes of *Aspergillus tamarii*

Variation source	SS	df	MS	*F*	Significance
Regression	2877.57386	3	959.1913	22.13452	*P* < 0.001
Residue	606.684969	14	43.33464		
Lack of fit	603.906	11			
Pure error	2.779	3			
Total	3484.259	17			

*r*² = 0.8258; *F*_3,14,0,01_ = 5.56. SS, square sum; df, degrees of freedom; MS, mean square.

The efficiency of enzymatic activity in any kind of process is always related to the application of optimum temperature and pH for enzyme activity. These parameters allow the highest reaction rate possible, ensuring the best results.

In the process of clarification of the broth *X. campestris* by crude extract enzyme produced by *A. tamarii*, under optimized conditions of cultivation, the reaction medium pH was not significant. This likely occurred because the crude enzymatic extract used contained several isoforms with proteolytic activity presenting different optimum pH of activity, as discussed earlier (Rao et al. 1998). This flexibility constitutes a major advantage for the industrial applicability of the process, as its effectiveness is independent of pH adjustment of the broth produced.

The optimum temperature for the process was determined in the range between 55 and 65°C, although satisfactory results have been obtained at temperatures up to 80°C, when in combination with higher concentrations of broth (Fig. 5). The foregoing is also a comparative advantage of the process for industrial application, since the enzymatic clarification can be performed concurrently or immediately after pasteurization of the broth, operation that normally occurs at temperatures between 65 and 75°C (García-Ochoa et al. 2000), so reducing the total processing time and the costs related to maintaining and adjusting the temperature of the broth.

Different studies (Triveni and Shamala 1999; Shastry and Prasad 2002) and patented processes (Patton 1976; Ernest and Wang 1981; Murofushi and Hawua 1998) for clarification of the culture broth of xanthan gum indicate dilution of the broth to ensure process efficiency. Possibly this is related to the viscosity of the broth and the difficulty of diffusion of lytic enzymes in the concentrated product. Shastry and Prasad (2002) studied the clarification of the broth in different dilutions (1:1–1:6) and obtained better results for final transmittance on the more diluted substrate. In the present investigation, the variable dilution of the broth showed a significant linear effect (*P* < 0.05), indicating that the higher the concentration of the broth used, the higher the transmittance obtained at the end of the reaction in clear disagreement with the work of Shastry and Prasad (2002)) and the general consensus. This unexpected behavior can be explained by the protective effect that the highest concentration of the broth seems to exert on the lytic activity of the enzyme extract. This can be verified by the observation of Figure 5, where it is apparent that at higher concentrations of the broth, higher transmittance values are reached, even at temperatures several degrees above optimum, possibly indicating that there is less thermal denaturation of the enzymes in these conditions. This is also an advantage of this process for industrial application, as there is no need to make any change to the culture broth obtained in the production of xanthan gum, not even dilution, to achieve satisfactory clarification.

## Conclusion

In accordance with the above exposition, it can be concluded that it was possible to produce high activity of lytic enzymes by cultivating the selected strain of *A. tamarii* by the solid-state fermentation process on medium consisting of coconut fiber with 90% humidity, supplemented with 2.5% gelatin and 30°C, after 20 days of incubation. The crude enzyme secreted under these conditions allowed the clarification of the culture broth of *X. campestris* for xanthan gum production, achieving transmittance of 70.6%, without dilution of the broth and without adjusting its pH at 65°C.
